# Behavioral Responses to Inequity in Reward Distribution and Working Effort in Crows and Ravens

**DOI:** 10.1371/journal.pone.0056885

**Published:** 2013-02-20

**Authors:** Claudia A. F. Wascher, Thomas Bugnyar

**Affiliations:** 1 Konrad Lorenz Forschungsstelle, Core Facility University of Vienna, Gruenau, Austria; 2 Department of Behavioural Biology, University of Vienna, Vienna, Austria; 3 Department of Cognitive Biology, University of Vienna, Vienna, Austria; Utrecht University, The Netherlands

## Abstract

Sensitivity to inequity is considered to be a crucial cognitive tool in the evolution of human cooperation. The ability has recently been shown also in primates and dogs, raising the question of an evolutionary basis of inequity aversion. We present first evidence that two bird species are sensitive to other individuals' efforts and payoffs. In a token exchange task we tested both behavioral responses to inequity in the quality of reward (preferred versus non-preferred food) and to the absence of reward in the presence of a rewarded partner, in 5 pairs of corvids (6 crows, 4 ravens). Birds decreased their exchange performance when the experimental partner received the reward as a gift, which indicates that they are sensitive to other individuals' working effort. They also decreased their exchange performance in the inequity compared with the equity condition. Notably, corvids refused to take the reward after a successful exchange more often in the inequity compared with the other conditions. Our findings indicate that awareness to other individuals' efforts and payoffs may evolve independently of phylogeny in systems with a given degree of social complexity.

## Introduction

The sensitivity regarding ones benefits of actions as well as efforts and payoffs for both the acting individual and its cooperation partner is proposed to be a key mechanism in the evolution of cooperation [1], [2], [3], [4]. Humans have been shown to have a strong preference regarding equity of reward distribution, also referred to as fairness [5]. They experience dissatisfaction when perceiving themselves under- as well as over-rewarded in experimental bargaining games [6] but also under more naturalistic conditions, e.g. job satisfaction [7], [8], [9]. Primates do behaviorally respond to disadvantageous inequity, i.e. situations in which an actor receives less than another individual even though both perform the same task ([10], [11], [12], [13]; but see [14], [15], [16]), suggesting an evolutionary basis of fairness in our close relatives [17]. Recently also dogs (*Canis familiaris*) have been shown to respond to inequity in the presence and absence of a reward [18], whereas no indication of inequity aversion (IA) could be found in cleaner fish (*Labroides dimidiatus*; [19]) indicating that forms of IA may be found in other mammals, but not in fish. What is yet unclear is how these findings fit into an evolutionary picture. Unlike primates, dogs do not respond to inequity in the quality of reward, raising the possibility that mammals other than primates possess a qualitatively different form of IA [18]. The more complex skills of primates would thus either build on the basic skills found in other mammals such as dogs or, in case the dogs derived their IA through domestication [20], would represent a unique feature of the primate linage. Alternatively, elements of IA may evolve independently of phylogeny in systems with a given cognitive complexity and, notably, specific demands of social life, i.e. iterated cooperative interactions with different individuals [17].

We here tested the latter assumption by expanding research on IA to large-brained birds. Specifically, we investigated sensitivity to unequal reward structure and working effort in two corvids, carrion crows (*Corvus corone corone*) and ravens (*Corvus corax*). Corvids have been shown to rival the cognitive abilities of primates in many respects, especially in the social domain [21], [22], [23], [24]. They may engage in various forms of naturally occurring cooperation [25], [26], [27] and demonstrate a high selectivity in partner choice in cooperative problem solving tasks [28], [29] as well as in coalition and alliance formation [30], [31]. We thus expect both crows and ravens to be sensitive to inequity in reward distribution not only on a yes-and-no basis shown by dogs, but on a more fine-grained basis similar to primates.

We tested the birds' performance in a token exchange task, comparable to that used by Brosnan and colleagues [10], [11], first rewarding subjects with differing food quality (task A) and then rewarding subjects versus not rewarding subjects for their action (task B). In the equity condition, both individuals are treated similarly by the human experimenter, whereas in the inequity condition, the focal individual receives a lower quality reward (task A) or no reward (task B) for the same action than the model individual. Critical for our interpretations are two further conditions: in the so-called effort control [10], [11], the model receives the reward as a ‘gift’ whilst the focal individual is asked to exchange, i.e. to ‘work’, for the same reward; note that this control is applied in both tasks (A, B) and can also be seen as test for another form of IA, working effort. In the quality control, the focal individual receives a low quality food reward for exchanging, but unlike the inequity condition, it is tested without a partner; the corresponding non-social control in task B is the no reward no partner condition. If corvids are averse in respect to inequity of reward distribution, their performance should drop in the inequity compared to the equity and quality control condition (task A) and equity, no reward no partner and both no reward condition (task B), respectively. If corvids are sensitive to other individuals working effort, their performance should drop in the effort control compared to the equity condition in both tasks.

## Methods

### Ethics Statement

**Figure 1:**
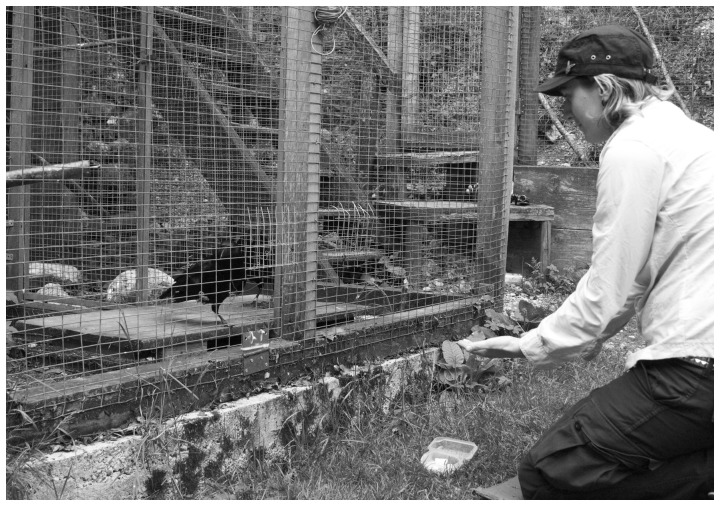
Photo of the experimental setup. Corvids stayed in adjacent experimental compartments, separated from each other by a wire mesh. The experimenter kneeled in front of the compartments, having both reward typed visible for the birds in front of her.

This study complied with Austrian and local government guidelines, permission to conduct non-invasive cognition experiments was received by CW from the head of the Konrad Lorenz research station (KLF), Prof. Kurt Kotrschal. As experiments were entirely non-invasive, no further animal experimental license was required (http://www.ris.bka.gv.at/GeltendeFassung.wxe?Abfrage=Bundesnormen&Gesetzesnummer=10010558). The experimenter (CW) has given written informed consent, as outlined in the PLOS consent form, to publication of her photograph (Fig. 1). Experiments have been conducted by an experienced animal caretaker, which ensures no animals to be harmed by the experimental procedure. All individuals participated and entered the experimental compartments voluntarily. Focal individuals are either zoo-bred (3 ravens) or rescued from the wild when taken out of the nest at a young age by unfavorable weather conditions (1 raven, crows). Individuals remain captivity housed in the Cumberland game park and the KLF (under the license AT00009917), before and after completion of the present study.

### Study subjects and testing procedure

Tests were conducted from January to July 2010 on six captive carrion crows (three males, three females) and four captive common ravens (three males, one female) at the KLF and the Cumberland game park, Austria. Individuals are housed in pairs in large outdoor aviaries and were tested in fixed dyads; four dyads consisted of affiliated pair partners/mates and one (raven) dyad consisted of non-affiliated but adjacently housed males. Crow dyads were tested individually in adjacent experimental compartments, separated from each other and the experimenter by a wire mesh (Fig. 1). Testing compartments in the raven dyads were not adjacent but separated by another compartment in which the experimenter stayed. Tested ravens were thus positioned two and four meters apart from another. In 2008, all birds were successfully trained to exchange a token (non-edible item) for food with a human experimenter [32], [33]. The present study is the first in which birds were confronted with a social setup. Dyads were tested in sessions of 24 trials, with at least 3 hours between sessions, and no more than 3 sessions per day. Each bird in a dyad was tested in both roles (focal and model) but only in one role within a session, with roles alternating every session. The focal was the first to exchange, followed by the model. Roles were initially allocated randomly. Each individual received 48 trials per condition. Task A has four conditions, therefore each dyad received 16 sessions in total, task B has five conditions, each dyad received a total of 20 sessions.

In each task, we performed two sessions per subject in each condition, with 24 trials per session. Grapes (1/4) were used as low quality food rewards and cheese (0.5 cm×0.5 cm×0.1 cm) as high quality reward in both tasks. Grapes are a regular part of the birds' daily diet, whereas cheese is exclusively used as rewards in experiments. In all conditions, both reward types were present and visible to the focal subjects, in two separate transparent containers. In the raven dyads, containers where placed one and two meters away from the birds, in the middle of the experimental compartment. In the crow dyads approximately 50 cm away from the birds placed directly in front of the experimenter (Fig. 1). In dyads tested beside each other, the experimenter knelt in front of the birds and was able to test both birds without moving from this position. In the raven dyads, the experimenter would exchange with one bird and then move to the other one. The experimenter initially presented the non-edible item (stone) in one open palm and the reward item (depending on the condition grape, cheese or nothing) in the other to the focal subject. This ensured that the birds could know for which reward they were asked to exchange and afterwards make their decision whether to exchange or not. The stone was offered to the subject by the experimenter for 3 seconds. This was repeated three times; if the subject did not take the stone into its beak then the trial was rated a ‘refuse to take the initial item’. If the subject took the stone, it then had to hold it in its beak for a minimum of two seconds before being asked to give the stone back. The subject was asked to give back the initial item into the open palm of the experimenter held close to the wire mesh. This was repeated three times for three seconds each. During this time, the reward stayed visible for the bird in the other (open) palm of the experimenter. In order to succeed, the focal subject had to place the stone in the experimenter's open palm; the experimenter then immediately passed the reward to the bird (for different failure types, Table 1). Individuals were allowed to eat the reward immediately, store it in their pouch or to cache it. We performed a maximum of three experimental sessions per day with at least three-hour breaks between sessions. All birds were tested first in task A (quality of the food reward) and afterwards in task B (absence of food reward). Individuals were tested in four (task A) and five (task B) experimental conditions (Table 2). The terminology used to describe experimental conditions is based on previous experiments in primates [10], [11]. The sequence of these conditions was counterbalanced, but initially started with the first equity condition to ensure that birds were aware of the experimental procedure in the social setting. In the two conditions of task A in which both subjects received the same type of reward (equity, effort control), we ensured that individuals received both reward types equally often (12 trials rewarded with cheese, 12 trials rewarded with grapes), in randomized order.

**Table 1:** Behavioral responses of crows and ravens during the exchange experiment

success	exchange	individual took the initial item with the beak and upon request gave it back into the hand of the experimenter
	refuses reward	individual successfully exchanged but refuses to take the reward
failure	refuses initial item	individual refuses to take the initial item
	drop	individual drops initial item but not into the hand of the experimenter
	does not give back	individual does not give back the initial item upon request

**Table 2:** Experimental conditions conducted in the present study.

condition	task	action focal	action model	reward focal	reward model	
equity	task A	exchange	exchange	cheese and grape	cheese and grape	
equity low quality	task B	exchange	exchange	grape	grape	
effort control	task A	exchange		cheese and grape	cheese and grape	
	task B			grape	grape	
quality control	task A	exchange	not present	grape		
no reward no partner	task B	exchange	not present	not rewarded		
both no reward	task B	exchange	exchange	not rewarded	not rewarded	
inequity	task A	exchange	exchange	grape		cheese
	task B	exchange	exchange	not rewarded		grape

### Statistical analysis

We used general linear mixed models (GLMMs) with binomial error distribution and a logit function. Response variables were the individual's behavioral response in each trial: exchange yes/no or refusal to take the reward. Condition, species, sex, dyad, session, start as focal or model, reward in the previous trial and type of reward (grape as low quality food, cheese as high quality food) served as fixed factors. In order to account for repeated measures for each individual, the individual identity was included as a random factor. All interactions between fixed factors as well as fixed factors and individual identity, remaining in the final model were included in the model. We selected the final model using a combination of a backward stepwise selection eliminating least significant factors from the model in order to reach the best model. This was determined using second order Akaike's information criteria (AICc), which compares the adequacy of several models and identifies the model which best explains the variance of the dependent variable as that with the lowest AICc value [34]. All factors and interactions remaining in the final model are presented here, irrespective of their significance. GLMM analyses were performed in SPSS 19.0. In unbalanced designs with more than one effect, the arithmetic mean for a group may not accurately reflect the response for that group, since it does not take other effects into account. Therefore, for *post-hoc* comparisons, we divided the differences between the parameter estimates by the standard error (SE) differences between pairs and interpreted the output as a t-test, with the degrees of freedom being equal to the residual of the model [35], [36]. All tests were two-tailed, with alpha set at 0.05. After Bonferroni correction to account for multiple testing, alpha in task A was set at 0.008 and alpha in task B at 0.01.

## Results

### Task A: Quality of reward

**Figure 2:**
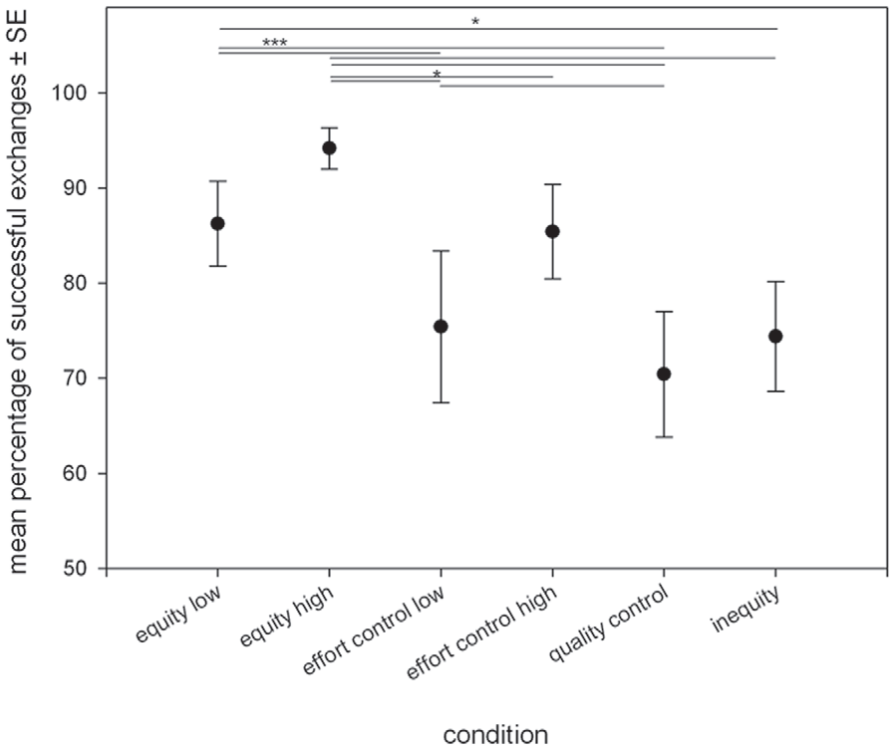
Corvids behavioral responses to task A, inequity in the quality of the food reward. Graph shows mean percentage of successful exchanges ± SE. ^*^p<0.05; ^**^p<0.01; ^***^p<0.001; Alpha after Bonferroni correction 0.0125.

Our final model included condition, reward in the previous trial, the quality of reward and the interactions between condition*individual and session*individual as significant predictors of success rate in exchanging with the human experimenter in task A (Table 3). Number of successful in each condition is presented in Table 4. *Post-hoc* comparisons between conditions revealed the following pattern (Fig. 2, Table 5):

**Table 3:** Factors and interactions remaining in the final model investigating exchange performance of corvids in response to inequity.

task A: inequity in reward quality					
fixed factors	numerator df	denominator df		F	p
condition	3	1.787		3.278	0.02
type of reward	1		1.787	11.769	0.001
reward in previous trial	1		1.787	7.206	0.007
session	1		1.787	2.557	0.11
				
interactions					
condition * individual	27		1.787	2.568	<0.001
session * individual	9		1.787	4.042	<0.001

task B: inequity in the absence of food reward				
session	1	2.335	16.617	<0.001
condition	4	2.335	5.369	<0.001
type of reward	1	2.335	0.011	0.74
interactions				
session*condition	4	2.335	7.073	<0.001
session*individual	9	2.335	9.969	<0.001
individual*condition	36	2.335	5.12	<0.001

Factors and interactions (in bold) significantly influence exchange performance.

**Table 4:** Number of successful exchanges and number of reward refusals (in brackets) in each condition.

	equity		effort control	quality control	inequity
	grape	cheese			
task A: inequity in reward quality					
Crows					
3109301^m^	23 (1)	23 (0)	48 (0)	34 (0)	38 (2)
HF54707^m^	19 (1)	23 (0)	39 (0)	34 (0)	35 (4)
HF54710^f^	19 (0)	22 (1)	34 (2)	44 (8)	28 (13)
HF54709^m^	16 (2)	19 (0)	30 (0)	19 (0)	19 (4)
HF54706^f^	20 (0)	22 (0)	48 (0)	25 (2)	43 (4)
HF54708^f^	15 (5)	21 (0)	19 (0)	22 (10)	38 (28)
Ravens					
JC46070^f^	23 (0)	24 (0)	41 (6)	34 (0)	37 (15)
JC38974^m^	24 (0)	24 (0)	44 (1)	46 (0)	46 (4)
JC38981^m^	24 (0)	24 (0)	35 (0)	32 (0)	27 (0)
JC46083^m^	24 (0)	24 (0)	48 (0)	48 (0)	46 (0)

task B: inequity in the absence of food reward					
	equity low quality	effort control	no reward, no partner	both no reward	inequity
Crows					
3109301^m^	43 (0)	35 (0)	18	10	24
HF54707^m^	43 (20)	34 (0)	35	41	44
HF54710^f^	11 (0)	3 (0)	8	24	20
HF54709^m^	18 (1)	22 (0)	5	24	9
HF54706^f^	30 (0)	13 (0)	18	17	35
HF54708^f^	34 (0)	32 (0)	4	14	19
Ravens					
JC46070^f^	25 (0)	22 (0)	21	13	6
JC38974^m^	26 (0)	1 (0)	14	10	8
JC38981^m^	20 (0)	33 (0)	33	24	16
JC46083^m^	44 (0)	46 (0)	29	19	12

Please note that in task B (inequity in the presence/absence of reward), reward refusals where not possible in the no reward, no partner, both no reward and inequity conditions, as focal individuals did not receive any reward for exchanging. In each condition each individual received a total of 48 trials (2 sessions á 24 trials). ^f^ female individuals, ^m^ male individuals.

**Table 5:** *Post-hoc* comparisons between different test conditions.

	T	p-value
**task A: inequity in reward quality**		
equity high-equity low	0.171	0.87
**equity high-effort control high**	**2.88**	**0.03, n.s. after Bonferroni**
**equity low-effort control low**	**4.14**	**<0.001**
**equity high-effort control low**	**2.88**	**0.03, n.s. after Bonferroni**
equity low-effort control high	1.72	0.14
**equity high quality reward-quality control**	**8.23**	**<0.001**
equity low quality reward-quality control		
**equity-inequity (high quality reward)**	**5.35**	**<0.001**
**equity-inequity (low quality reward)**	**2.61**	**0.04, n.s. after Bonferroni**
effort control high-effort control low	2.38	0.06
effort control low-quality control	0.32	0.76
effort control low-inequity	1.03	0.35
effort control high-inequity	1.6	0.17
inequity-quality control	1.826	0.12
**task B: inequity in the absence of food reward**		
**equity-effort control**	**2.62**	**0.039, n.s. after Bonferroni**
**equity-no reward no partner**	**4.44**	**0.004**
**equity-both no reward**	**3.65**	**0.01**
**equity-inequity**	**3.74**	**0.009**
effort control-no reward no partner	2.37	0.055
effort control-both no reward	1.56	0.169
effort control-inequity	1.65	0.15
no reward no partner-both no reward no partner	0.93	0.388
no reward no partner-inequity	0.83	0.438
both no reward- inequity	0.87	0.417

Differences between the parameter estimates have been divided by the standard error (SE) differences between pairs and interpreted the output as a t-test.

According to our expectation concerning reward distribution, exchange rate was higher in the equity condition than in the inequity condition, whereby the effect was stronger with high quality food (T = 5.35, p<0.001) than with low quality food (T = 2.61, p = 0.04; n.s. after Bonferroni). According to our expectation concerning sensitivity to effort, exchange rate was higher in the equity condition than in the effort control, whereby the effect was smaller with high quality food (T = 2.88, p = 0.03; n.s. after Bonferroni) than with low quality food (T = 4.14, p<0.001; see Table 5 for further results).

**Figure 3:**
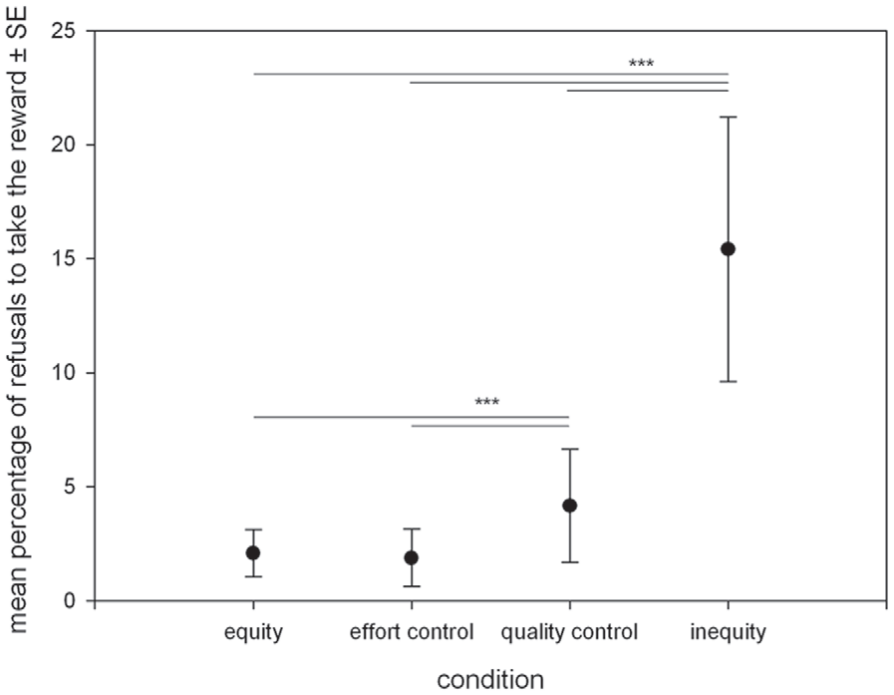
Mean percentage ± SE of successful exchanges with the experimenter but refusals to take the offered reward in task A. Equity- effort control (T = 0, p = 1); equity- quality control (T = 7, p = 0.001); equity-inequity (T = 10.5, p = 0.001); effort control- quality control (T = 7.5, p = 0.001); effort control- inequity (T = 10.6, p = 0.001); quality control-inequity (T = 12.25, p = 0.01).

Figure 3 denotes the pronounced occurrence of a particularly interesting refusal type in task A: when the focal individual successfully exchanged the initial item, but then refused to take the reward. In contrast to other types of behavioral responses (Table 1), here the focal individual ended up without anything, neither the initial object nor the food reward. Focal individuals showed this behavior significantly more often in the inequity condition (GLMM: df_1_ = 4, df_2_ = 1.88, F = 234.49, p = 0.001) and the interaction between condition*individuals was significant (GLMM: df_1_ = 36, df_2_ = 1.88, F = 1.905, p = 0.001).

### Task B: Absence of reward

**Figure 4:**
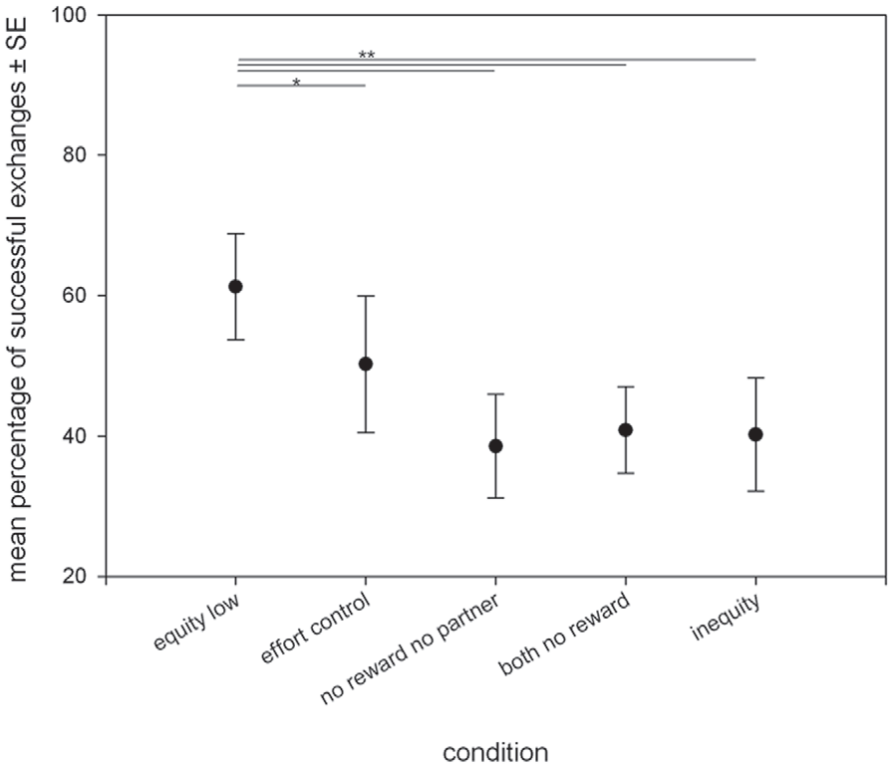
Corvids behavioral responses in experiment B exchange in the absence of a reward. Graph shows mean percentage of successful exchanges ± SE. Alpha after Bonferroni correction 0.01.

In task B, condition significantly influenced exchange performance (Table 1, 4, Fig. 4). Besides condition, the interactions between session*individual, session*condition, individual*type of reward and individual*condition also had a significant effect on exchange performance. Session and the type of reward remained in the final model but did not have a significant effect.

In conditions where the focal individual did not receive any reward for exchanging, performance dropped dramatically compared to the equity condition (equity- no reward no partner T = 4.44, p = 0.004; equity-both no reward T = 3.65, p = 0.01; equity- inequity T = 3.74, p = 0.009; see Table 5 for details on non-significant comparisons). This was expected for the inequity condition but not (as much) for the other conditions. Concerning sensitivity to effort, our prediction held: birds tended to exchange less in the effort control compared to the equity condition (T = 2.62, p = 0.039, n.s. after Bonferroni).

## Discussion

To our knowledge, this is the first evidence of behavioral responses to inequity in birds. As expected, crows and ravens responded to inequity in outcome, i.e. to difference in quality (task A) and to presence/absence of the reward (task B); moreover, they were highly sensitive to inequity in working effort in both tasks. In addition, the quality of the offered food (grapes and cheese) influenced the birds' exchange performance, whereby the differences to inequity and quality were most pronounced with highly valued food (cheese).

Interestingly, the strongest effect of the current study was found in respect to working effort: when the model individual received the reward as a ‘gift’ without exchanging a token first, the focal bird, required to exchange for the reward, decreased its performance. These results are of special interest, as a main criticism regarding inequity aversion (IA) in non-human animals is that their behavioral responses might reflect individual frustration and/or response to negative contrast (i.e. receiving less preferred food after receiving more preferred food) rather than sensitivity to inequity [14], [37]. Indeed, the pronounced effects of food quality in the current study may be seen as support for this argument. Note that this critique does *not* apply to the current results on *working effort* because individuals received the same reward types in the two critical conditions, equity and effort control, with working effort being the only difference between focal and model individuals. Possibly witnessing another individual receiving food may make the focal bird expect to be given such a piece of food too, and thus makes it less likely to exchange. Still, there are striking differences in the sensitivity to working effort across species. The responses of corvids found here appear to be stronger than those reported for great apes [38], [39], [40] but similar to those of some monkeys [11], [39]; the study on dogs [18], however, has not found any evidence at all.

Regarding inequity in the outcome, corvids decreased their exchange performance in the inequity, compared to the equity condition in both tasks. However, in task A, it is difficult to disentangle the effects of food quality and inequity. Exchange rates in the inequity condition are not significantly different to rates in the quality control, where individuals were tested alone and received a low quality food reward for exchanging. Refusals in the inequity condition could thus simply be due to the low quality of the offered reward.

The fact that crows and ravens did respond to the non-social controls of tasks A and B with a drop in performance underlines that they are highly sensitive to the social context. Indeed, the mere presence of a social partner seemed to have a facilitating effect on the birds' motivation to exchange. In parallel studies using the same paradigm, we could perform eight trials per sessions when individuals were tested alone [32], which is only a third of the trials the birds were willing to do per session in this study. Thus, a social set-up may have generally boosted their propensity to exchange, confounding possible effects between quality control and inequity in task A. Moreover, the pronounced occurrence of a specific form of refusal, namely rejecting the reward after successfully completing the exchange in the inequity condition provides further support that the birds did treat inequity in reward differently from low food quality.

To control for a possible frustration effect in our tasks, the prospected reward was always presented to both, the model and the focal individual in advance of the exchange. Therefore, individuals were expected to refuse to exchange when treated unequal, but not to complete the task and then refuse the reward. Refusing the reward after exchanging the initial item presents a clear cost, as individuals are outputting the working effort but not accepting the reward. That the exchange task reflects some working effort for corvids is illustrated by the birds' behavior in the effort control condition of the current study. Moreover, in another study, crows preferred larger quantities of food over smaller ones in a choice task, but were not willing to exchange one piece of food for a larger number of the same reward [33]. Finally, we know from ravens that they may put high value to objects others are interested in, protecting them from being stolen [41], [42]. Hence, it is unusual that they give away tokens without accepting to get anything in return. We thus conclude that they reject unfair offers even at a cost to themselves [3]. In primates, the finding that they refuse more in the inequity condition has been suggested as an indication of disadvantageous IA. These higher refusal rates are absent in dogs, which will delay participation but ultimately do not refuse [18]. Although the results of the present study are comparable to previous studies in many respects [11], [17], [39], we need to be cautious with interpreting them as evidence for IA in corvids. We certainly found strong indications for a facilitating effect of reward equity and working effort onto exchange performance. If the underlying cognitive mechanism is IA has to be shown in further studies.

A limitation of the presented study is the low sample size. As only six crows and fours ravens were available for testing, we must be cautious with generalizing our findings. For instance, it might be possible that species differences between crows and ravens exist, but could not be detected with our sample. Also, the interactions between fixed factors (condition, session) and individual identity indicates that individuals do respond differently to inequity. For example regarding the significant interaction between session*individual, most birds increased performance in the course of the experiment, except two which decreased exchange rates in later sessions. This could be due to many factors such age, sex, affiliation status and ‘personality’ [38]. To draw firm conclusions about the evolution of IA in systems with a type of social complexity typical for crows and ravens, further investigations in a larger sample, other populations and other closely related corvid species would be desirable.

Despite those limitations, we would like to stress that the birds in our study performed qualitatively similarly to some primates [11], [17], [39], highlighting the fact that differences in sensitivity to reward distribution and working effort can be more pronounced among mammals (i.e. primates and dogs, [18]) than between corvids and primates. Recent evidence within the primate order suggests convergent rather than homologous evolution of IA [43] and has been linked to cooperative tendencies within species [17]. In crows and ravens, various forms of naturally occurring cooperation have been described under both field and captive conditions (e.g. cooperative breeding, alliance formation, food and information sharing: [23], [25], [26]); however, little is known about the mechanisms underlying their cooperation. Recent studies suggest that cooperative interactions occur quite flexibly among related as well as unrelated individuals [30], [44] and on a reciprocal basis between long-term and, possibly, short-term interaction partners [30], [31]. Similar to primates, a high flexibility in cooperation may have driven the evolution of sensitivity to other individuals' efforts and payoffs in corvids. Applying the comparative approach to systems with different degrees of complexity but independently of phylogeny may be the key for testing these assumptions.
